# Rapid diagnosis of celiac disease based on plasma Raman spectroscopy combined with deep learning

**DOI:** 10.1038/s41598-024-64621-4

**Published:** 2024-07-01

**Authors:** Tian Shi, Jiahe Li, Na Li, Cheng Chen, Chen Chen, Chenjie Chang, Shenglong Xue, Weidong Liu, Ainur Maimaiti Reyim, Feng Gao, Xiaoyi Lv

**Affiliations:** 1 Department of Gastroenterology, People’s Hospital of Xinjiang Uygur Autonomous Region, Urumqi, 830001 Xinjiang Uygur Autonomous Region China; 2 College of Software, Xinjiang University, Urumqi, 830046 China; 3 Present Address: Xinjiang Clinical Research Center for Digestive Diseases, No. 91 Tianchi Road, Tianshan District, Urumqi, 830001 Xinjiang Uygur Autonomous Region China

**Keywords:** Celiac disease, Plasma, Raman spectroscopy, Machine learning, Biological techniques, Diseases

## Abstract

Celiac Disease (CD) is a primary malabsorption syndrome resulting from the interplay of genetic, immune, and dietary factors. CD negatively impacts daily activities and may lead to conditions such as osteoporosis, malignancies in the small intestine, ulcerative jejunitis, and enteritis, ultimately causing severe malnutrition. Therefore, an effective and rapid differentiation between healthy individuals and those with celiac disease is crucial for early diagnosis and treatment. This study utilizes Raman spectroscopy combined with deep learning models to achieve a non-invasive, rapid, and accurate diagnostic method for celiac disease and healthy controls. A total of 59 plasma samples, comprising 29 celiac disease cases and 30 healthy controls, were collected for experimental purposes. Convolutional Neural Network (CNN), Multi-Scale Convolutional Neural Network (MCNN), Residual Network (ResNet), and Deep Residual Shrinkage Network (DRSN) classification models were employed. The accuracy rates for these models were found to be 86.67%, 90.76%, 86.67% and 95.00%, respectively. Comparative validation results revealed that the DRSN model exhibited the best performance, with an AUC value and accuracy of 97.60% and 95%, respectively. This confirms the superiority of Raman spectroscopy combined with deep learning in the diagnosis of celiac disease.

## Introduction

Celiac Disease (CD) is an autoimmune digestive system disorder characterized by impaired fat digestion or absorption, resulting in the excretion of substantial amounts of fat and giving stools a milky appearance^1^. Under normal circumstances, the digestive system efficiently breaks down fats, allowing for absorption and transportation into the body. However, in CD patients, this process is disrupted, preventing adequate fat absorption. The presence of large amounts of unabsorbed fats in the intestines can lead to irritation, potentially causing diarrhea. Additionally, the loss of essential nutrients such as fats, proteins, and fat-soluble vitamins may result in malnutrition, leading to various health issues^2^. CD can impact growth and development and compromise the immune system, increasing the risk of infections and other diseases. Individuals with CD commonly experience gastrointestinal symptoms such as diarrhea, abdominal pain, and bloating, significantly affecting their quality of life^3^.

Research indicates that early diagnosis and treatment of the disease can effectively slow down its progression. Therefore, establishing a rapid and accurate diagnostic method is of paramount importance for achieving early detection of CD and reducing associated damages^4^. Currently, the diagnosis and classification of CD depend on factors such as patient medical history, physical examinations, laboratory findings, and radiological evidence^5^. Diagnostic methods often involve examining fecal fat content^6^ and conducting blood and intestinal mucosal biopsies^7^. However, diagnosing CD remains challenging due to its symptoms being subtle or mistaken for other gastrointestinal issues. The most common reasons for CD screening include abdominal bloating and diarrhea, but these symptoms may not always be evident. This difficulty in diagnosis can lead to delayed or incorrect treatment, exacerbated by significant variations in the course of CD among patients. Some may exhibit mild symptoms, while others experience noticeable clinical manifestations, adding complexity to accurate diagnosis^8,9^. Early detection of CD is crucial, as timely intervention and treatment can significantly improve patients' quality of life and slow down disease progression.

As a rapid spectral analysis technique, Raman spectroscopy (RS) can measure various biomolecules present in plasma samples, including proteins, nucleic acids, carbohydrates, and lipids^10^. Intensity differences between Raman peaks are primarily attributed to nucleic acids, amino acids, and lipids, which play crucial roles in biochemical reactions such as biological transformations, immune process monitoring, signal transduction, and nutrient metabolism. Raman spectroscopy’s ability to capture a wealth of information from multiple peaks, each representing specific substances and their intensities and positions, makes it the “biological fingerprint” region of sample Raman spectra^11^.

Despite the significant achievements of Raman spectroscopy combined with machine learning models in disease diagnosis^12^, the technique has limitations, such as a low signal-to-noise ratio^11^. This limitation may hinder the intuitive identification of differences between spectra, potentially resulting in lower diagnostic performance. Therefore, exploring spectral differences through intelligent methods holds substantial significance.

Various chemometric techniques, including Principal Component Analysis (PCA), Support Vector Machine (SVM), and K-Nearest Neighbors (KNN), have been extensively applied in spectral analysis^13,14^. However, for extracting more features and achieving diagnostic requirements, more complex deep learning models are needed. Convolutional Neural Network (CNN) is one of the most popular foundational deep learning frameworks, reducing parameter numbers and improving feature extraction quality through local connections and parameter sharing. Traditional CNN models have demonstrated high performance and robustness in processing raw spectral data.

Studies by Yang et al.^15^ and Wu et al.^16^ showcase the effectiveness of one-dimensional CNNs in accurately classifying plasma lesions of tongue squamous cell carcinoma and rapidly diagnosing sparganosis using plasma Raman spectra, achieving accuracies of 94.90%. However, traditional CNN models can only extract local features at one scale, and the spectral measurement process is often susceptible to strong noise interference, making high-quality feature extraction more challenging. Additionally, differences arising from noise between training and testing sets may decrease spectral classification accuracy.

To address these challenges, this study constructs and adopts four different neural network models: CNN, Multi-Scale Convolutional Neural Network (MCNN), Residual Network (ResNet), and Deep Residual Shrinkage Network (DRSN). These models are trained end-to-end, eliminating the tedious process of manually extracting features. The models can automatically learn critical features from spectral data, enhancing generalization capabilities. By extracting features at different scales, the models effectively capture local information within the spectra, crucial for diagnosing celiac disease. Moreover, these models reduce noise interference during spectral measurement, further enhancing the neural network models' generalization capabilities and diagnostic accuracy for celiac disease.

## Materials and methods

### Experimental materials

In this study, we employed a pipette to collect 50 μL of plasma samples on tin foil-coated slides. After being partially dried in the air at room temperature (22 °C), but not completely dry, data were collected using a high-resolution confocal Raman spectrometer (Gora Raman Spectroscopy, Ideaoptics, China). The excitation wavelength was 785 nm from a YAG laser, 15-s integration time, and laser power of 160 mW. Continuous acquisition mode was set to measure the Raman spectra of plasma samples in the range of 500–2500 cm^−1^. The laser beam was focused on the sample surface through a 50× lens, and three Raman spectra data were measured for each individual sample. Other spectral measurement conditions included an 8-s integration time, three integrations, five iterations, and 64 baseline points. And the spectral resolution of our spectrometer is 6.06 cm^−1^. The celiac disease dataset tests three times at different positions on each sample. The study included 30 healthy control samples and 29 celiac disease samples, each measured three times, resulting in a total of 90 spectra for the control group and 87 for celiac disease.

**Figure 1:**
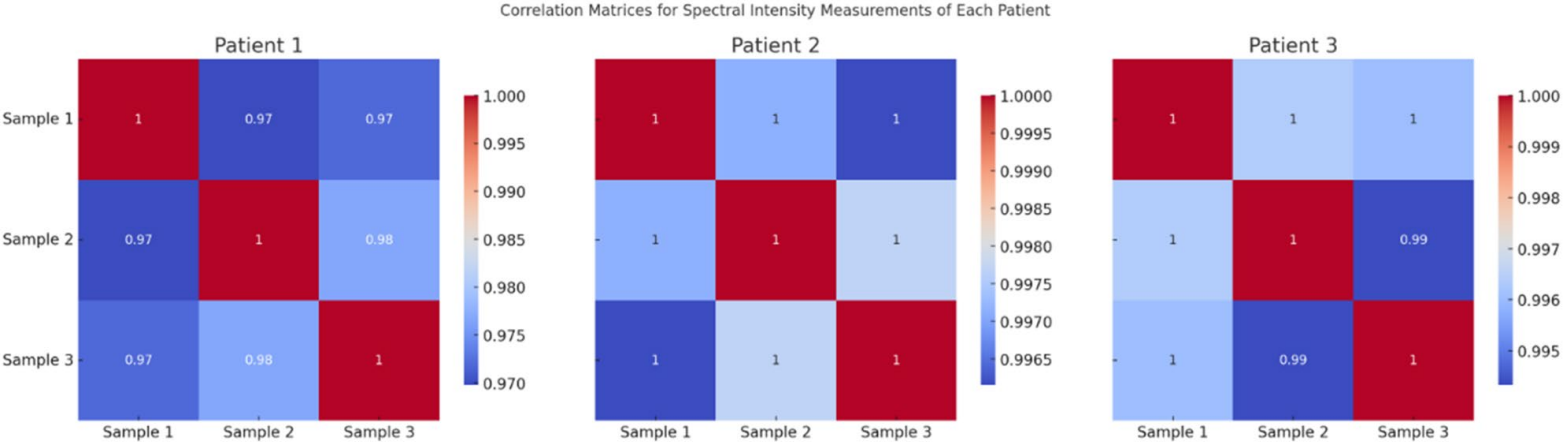
Correlation matrices for spectral intensity measurements of each patient.

**Figure 2:**
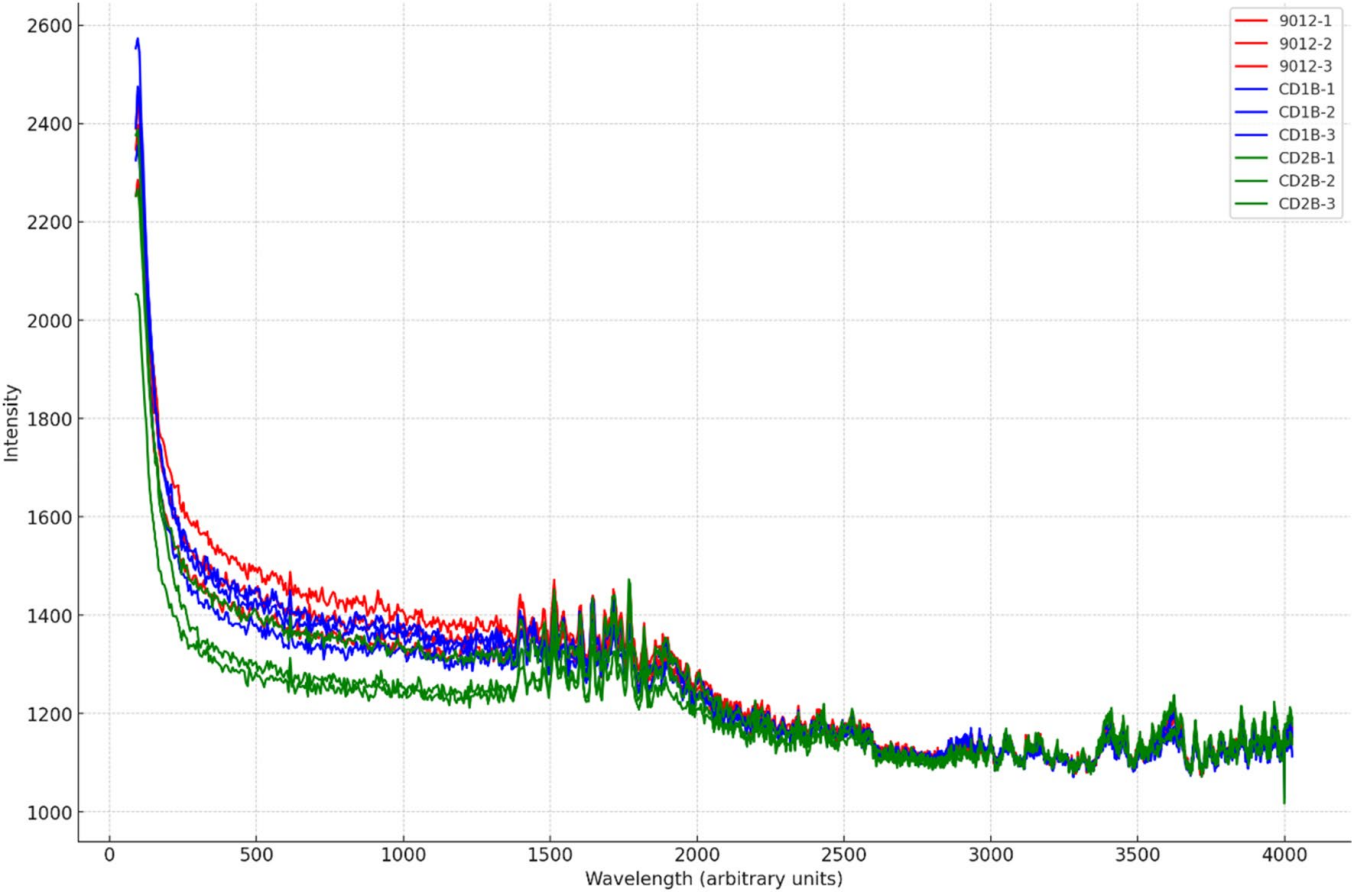
Randomly selected spectral intensity measurements across wavelengths for three patients.

In this study, the data were divided into training and testing sets. Since three spectra were collected for each patient sample, we averaged the spectra for each patient sample and conducted experiments using the averaged spectra. Therefore, after dividing the data into training and testing sets, we can ensure that the spectra of the same patient sample do not simultaneously exist in both the training and testing sets. All plasma samples were taken from fresh blood samples. The samples did not contain any anticoagulants and were collected in the morning after fasting for at least 12 h to avoid interference from dietary and other factors on the blood plasma components. We conducted correlation analysis of three samples from the same patient using the Pearson coefficient. We observed that the correlation between different patients has reached 0.99. The Pearson correlation coefficient for the correlation analysis among three samples of patient shown in the Fig. 1. The spectral intensity measurements across wavelengths for three randomly selected patients are shown in Fig. 2.

All samples were provided by the Autonomous Region People’s Hospital. The research protocol was approved by the Ethics Committee of the Autonomous Region People's Hospital (Approval Number: [(KY2023968173)]), and pathological examination of plasma confirmed the presence of Celiac Disease.

### Data preprocessing

The airPLS algorithm was applied to remove background signals from the Raman spectra^17^, followed by the implementation of the Smoothing algorithm for noise elimination^18^. In the Smoothing algorithm, a window length of 5 and a polynomial order of 2 were utilized. Before normalization, to eliminate noise in the spectrum, we employed Fourier transformation for low-frequency filtering to remove the low-frequency components.

The train_test_split() method from the sklearn standard library was employed to partition the preprocessed Raman spectroscopy data into a training set and a testing set, with a ratio of 7:3. The training set consisted of 40 spectral data, while the testing set comprised 19 spectral data. Five-fold cross-validation was performed on the classification model, and the testing set results were used as the final evaluation metrics.

### Model evaluation metrics

This study comprehensively assessed the performance of each model in the celiac disease classification diagnosis task using four parameters: accuracy, specificity, sensitivity, and precision. Accuracy represents the percentage of correctly predicted samples out of the total sample count, and the accuracy formula is as follows:

1 $$\begin{array}{*{20}c} {{\text{Accuracy}} = \left( {TP + TN} \right)/\left( {TP + TN + FP + FN} \right)} \\ \end{array}$$

where TP represents the count of samples correctly classified as positive in the positive sample class. TN represents the count of samples correctly classified as negative in the negative sample class. FP represents the count of samples incorrectly classified as positive in the negative sample class. FN represents the count of samples incorrectly classified as negative in the positive sample class. The formula for specificity is as follows:

2 $$\begin{array}{*{20}c} {{\text{Specificity}} = TN/\left( {TN + FN} \right) } \\ \end{array}$$

The formula for sensitivity is as follows:

3 $$\begin{array}{*{20}c} {{\text{Sensitivity}} = TP/\left( {TP + FN} \right) } \\ \end{array}$$

The precision formula is as follows:

4 $$\begin{array}{*{20}c} {{\text{Precision}} = {\text{TP/}}\left( {{\text{TP}} + {\text{FP}}} \right)} \\ \end{array}$$

AUC (Area Under the Curve) is a commonly used metric for assessing the performance of binary classification models. The AUC value represents the area between the True Positive Rate (also known as sensitivity or recall) and the False Positive Rate at different thresholds. It measures the model's ability to correctly distinguish between positive and negative instances at various classification thresholds. The typical AUC curve is the Receiver Operating Characteristic (ROC) curve, which plots the True Positive Rate against the False Positive Rate, illustrating the model's performance across different classification thresholds. A higher AUC value, closer to 1, indicates better classification performance, while a value close to 0.5 suggests that the model's performance is similar to random guessing. The basic structure of the confusion matrix is shown in Table 1.

**Table 1:** RS and SERS peak positions and vibrational mode assignments.

	Predicted positive	Predicted negative
Actual positive	True positive (TP)	False negative (FN)
Actual negative	False positive (FP)	True negative (TN)

### Ethics statement

All experiments in this study were performed in strict accordance with relevant principles, regulations, and guidelines. All samples were provided by the People's Hospital of the Autonomous Region. The research protocol received approval from the Ethics Committee of the People's Hospital of the Autonomous Region (Approval Number: (KY2023968173)).This study confirmed that informed consent was obtained from all subjects and/or their legal guardians.

## Results and analysis

### Principal component analysis and interpretability

**Figure 3:**
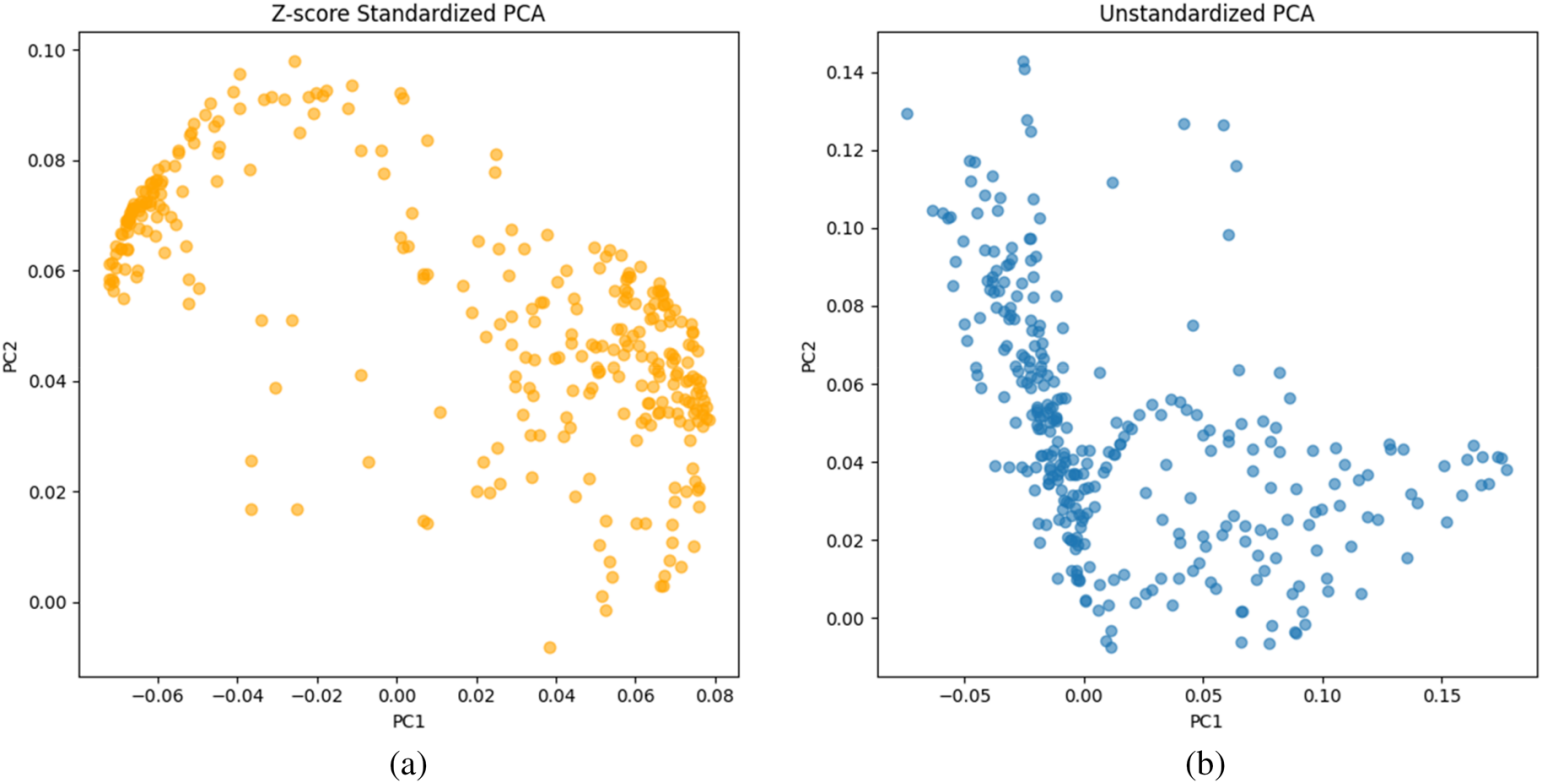
(**a**) PCA after Z-score standardization; (**b**) PCA without Z-score standardization.

Considering the characteristics of the dataset and the requirements of the analysis task, for Raman spectral data, we retained 85% of the explained total variance in the principal components. Meanwhile, we used the explained variance ratio attribute to obtain the variance explained by each principal component. We standardized the spectral data using z-score and combined PCA to visualize the data distribution (as shown in Fig. 3), providing a clearer depiction of how the data spread in terms of spectral standard deviation and principal component analysis.

**Figure 4:**
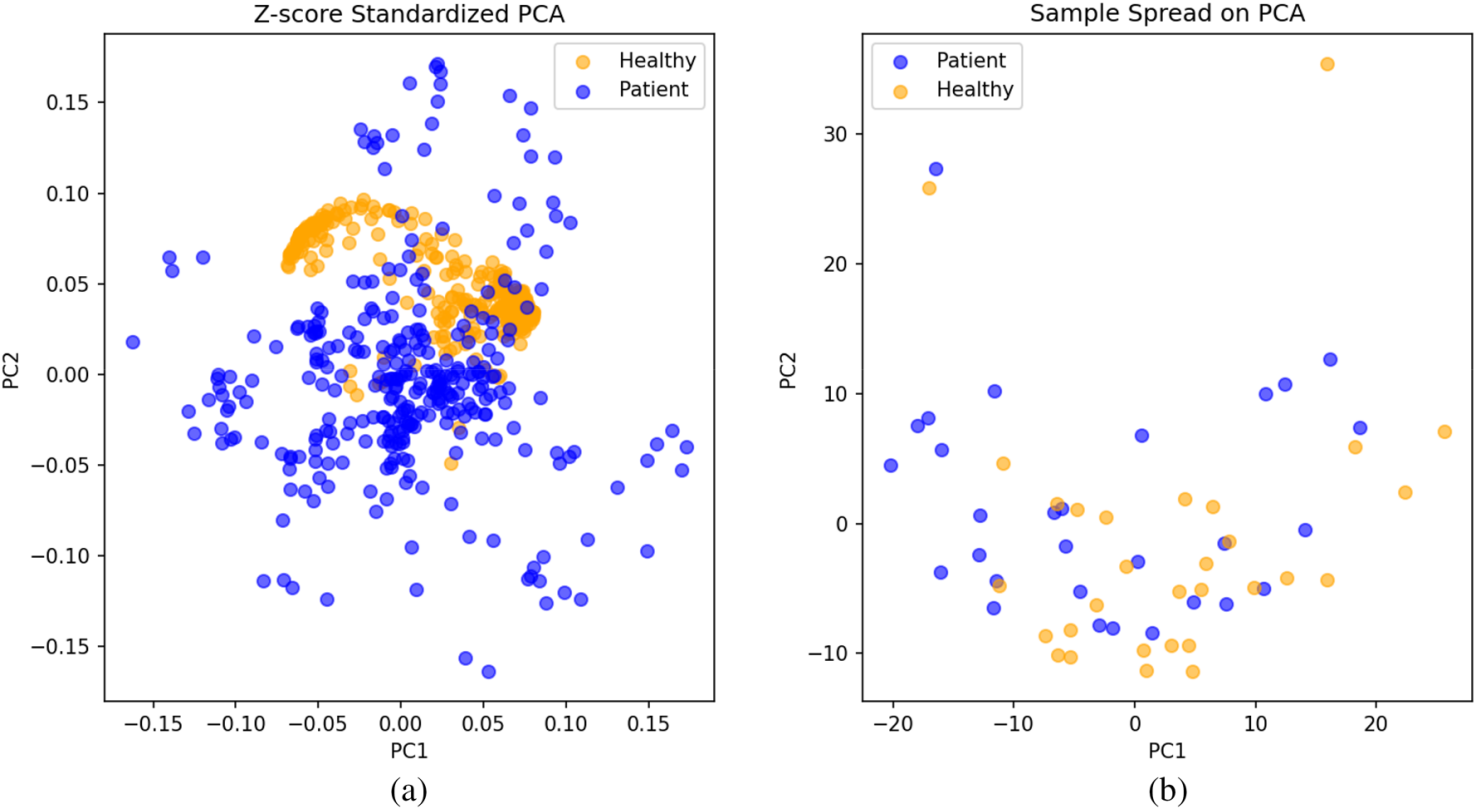
(**a**) Projection direction of data in the principal component space; (**b**) Projection of each sample in the principal component space.

Figure 4a illustrates the projection direction of the data in the principal component space. Specifically, each point represents a feature (in this case, possibly the original features or PCA components), rather than a sample. The coordinates of each point represent the weights on the corresponding principal components. Therefore, the left plot helps us understand the contribution of original features to the principal components and the relationships between the components. Figure 4b displays the projection of each sample in the principal component space. Each point represents a sample, and its coordinates are its projection values on the principal components. This plot helps us observe the distribution of samples along the principal component directions and the distinctiveness between different categories.

**Figure 5:**
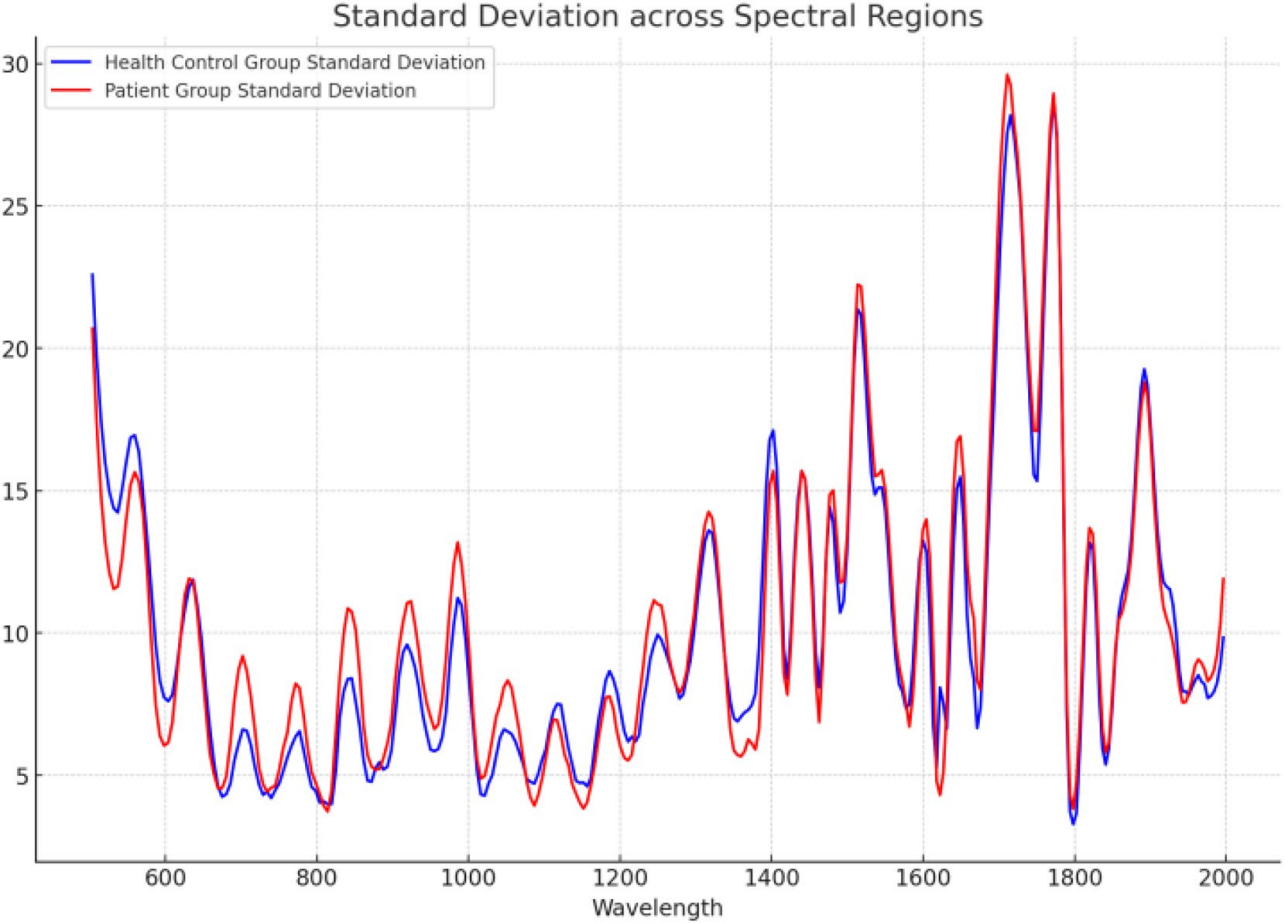
Standard deviation across spectral regions.

To visualize the variance of the entire dataset, we plotted a graph (as shown in Fig. 5) displaying the standard deviation of the entire dataset.

**Figure 6:**
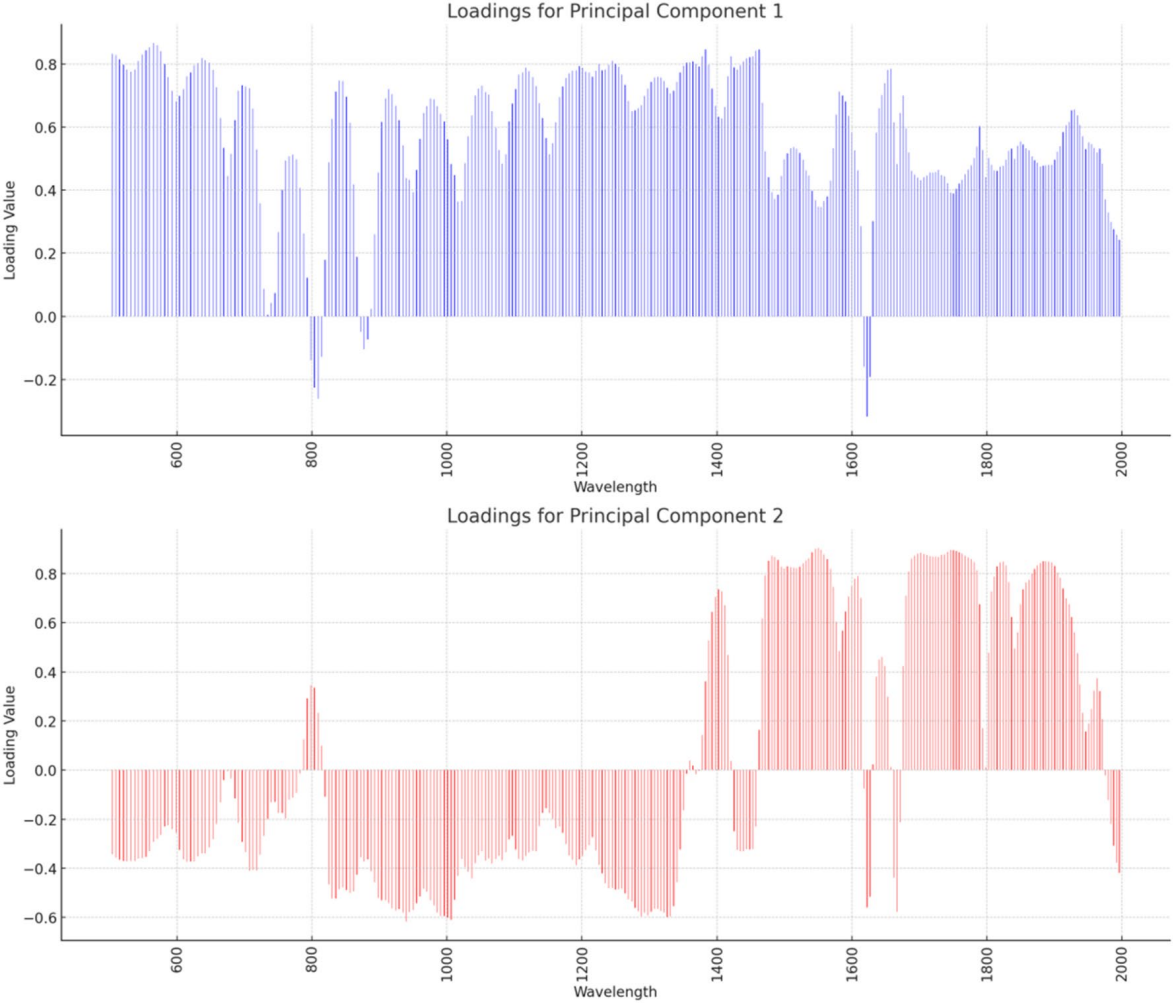
The contribution of each wavelength corresponding to the two principal components.

In spectral analysis, each wavelength corresponds to a feature. The spectral standard deviation in Fig. 5 reflects the dispersion of data at each wavelength. By showcasing the distribution of spectral standard deviation, we can understand the variability of data at different wavelengths and the dispersion of data across the spectral range. This helps illustrate how each sample in the dataset spreads in terms of spectral standard deviation and principal component analysis. The contributions of each wavelength to the two principal components are shown in Fig. 6.

Through the aforementioned visualizations, we can observe the variation and distribution of data across different feature dimensions. However, for the utilization of advanced classification tools such as neural networks, more data features are required for training and classification. Therefore, we can see that the distribution of data after PCA is not ideal, which serves as one of the reasons for choosing more advanced classification tools like deep learning models.

### Raman spectroscopy

**Figure 7:**
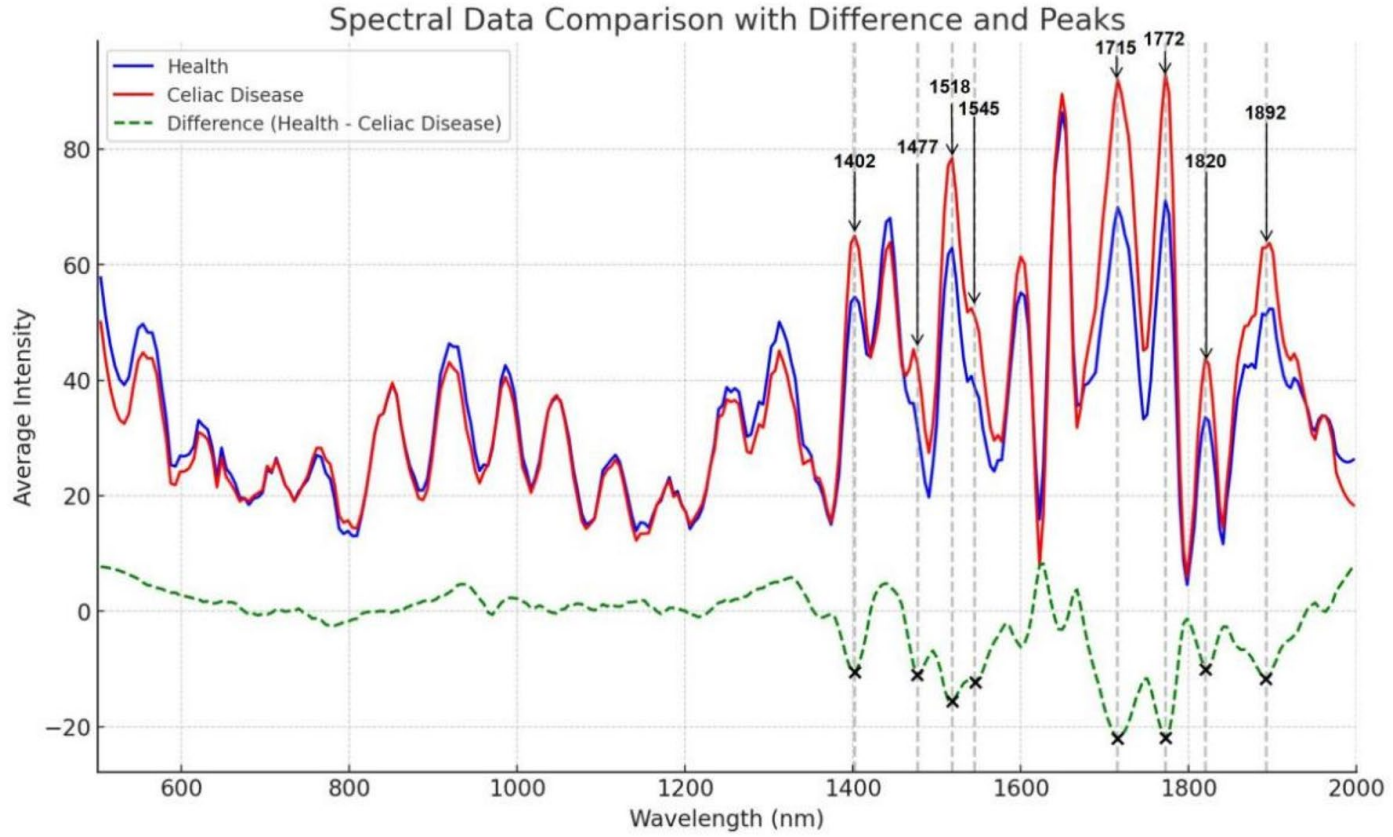
Average Raman spectra of Celiac Disease and healthy controls.

The Raman spectra of plasma from patients with celiac disease (CD) are shown in Fig. 1, where the Raman characteristic peaks represent substances rich in lipids, proteins, nucleic acids, and amino acids in the tissue. Previous studies have indicated that changes in Raman peaks of proteins and nucleic acids may be observed in the plasma of diseased individuals, reflecting abnormal expression of cellular nucleic acids and proteins^18^. Additionally, CD patients exhibit higher levels of high-sensitivity C-reactive protein in their plasma, and in terms of the lipoprotein spectrum, CD patients show lower levels of high-density lipoprotein cholesterol (HDL-C)^19^. The serum of CD patients is characterized by lower levels of various metabolites (such as amino acids, lipids, ketones, and choline) (P < 0.01)^20^. Comparative experiments have revealed that, in terms of lipids, the main differences between celiac disease patients and the control group are a decrease in cholesterol and phospholipids in both high-density lipoprotein and low-density lipoprotein in the former. These differences persist after treatment, and a lower level of cholesterol in very-low-density lipoprotein (VLDL) has also been observed^21^. Table 1 lists the major characteristic peaks of plasma in celiac disease, along with the assignment of each feature peak. As shown in Fig. 7, patients with celiac disease exhibit Raman peaks at 1402 cm^−1^, 1477 cm^−1^, 1518 cm^−1^, 1545 cm^−1^, 1715 cm^−1^ and 1772 cm^−1^, in their plasma, which are higher than those in normal controls. However, the peak at 1445 cm^−1^ is lower than in normal controls. Significant differences exist between celiac disease patients and healthy controls in terms of functionality, tissue structure, and surface features in plasma. Specifically, the notable Raman peak difference at 1402 cm^−1^ reflects differences in bending modes of methyl groups between the two groups, indicating potential abnormal lipid metabolism in celiac disease patients, such as damage to adipose tissue due to malabsorption of fat^22^. As shown in Table 2, The Raman peak at 1477 cm^−1^ reflects calcium oxalate in the patient's plasma, exhibiting significant changes compared to healthy plasma^23^. Celiac disease is an immune-related disease that may involve an abnormal immune response to proteins in the intestines. This may lead to observed Raman peak differences in celiac disease patients, reflecting changes in protein structure or composition. In celiac patients, changes in lipid and protein composition are related to alterations in cell membrane structure and function due to damage to the intestinal mucosa. Additionally, celiac disease is often accompanied by inflammation and the formation of immune complexes. These biological processes may cause changes in the intra- and extracellular environment, including the distribution and structure of lipids and proteins. The Raman peak difference at 1518 cm^−1^ is attributed to differences in cytosine content. In celiac patients, the impact on nucleotides, including changes in concentration or structure, may occur due to intestinal damage. The expression level changes of phenylalanine are reflected in the Raman peak at 1545 cm^−1^, indicating the metabolic status, redox balance, and regulation of some physiological functions. Differences in the Raman spectrum of C=O vibration at 1715 cm^−1^ and 1772 cm^−1^ are lipid-related, as celiac disease is a malabsorption disease. Therefore, if significant differences in C=O vibration are detected in celiac patients, it implies abnormal lipid metabolism or changes in lipid composition, which are related to the absorption and metabolism of fat in the intestines^22^.

**Table 2:** The major Raman bands and their corresponding assignments^35^.

Wavenumber (cm^−1^)	Corresponding substance	RS
1402	Bending modes of methyl groups	√
1477	Calcium oxalate	√
1518	Cytosine	√
1545	Bound and free NADH 76 Tryptophan	√
1715	C=O lipids	√
1772	C=O	√

√: Presence of related substances;

### Model evaluation

#### Convolutional neural network (CNN) model evaluation

**Figure 8:**
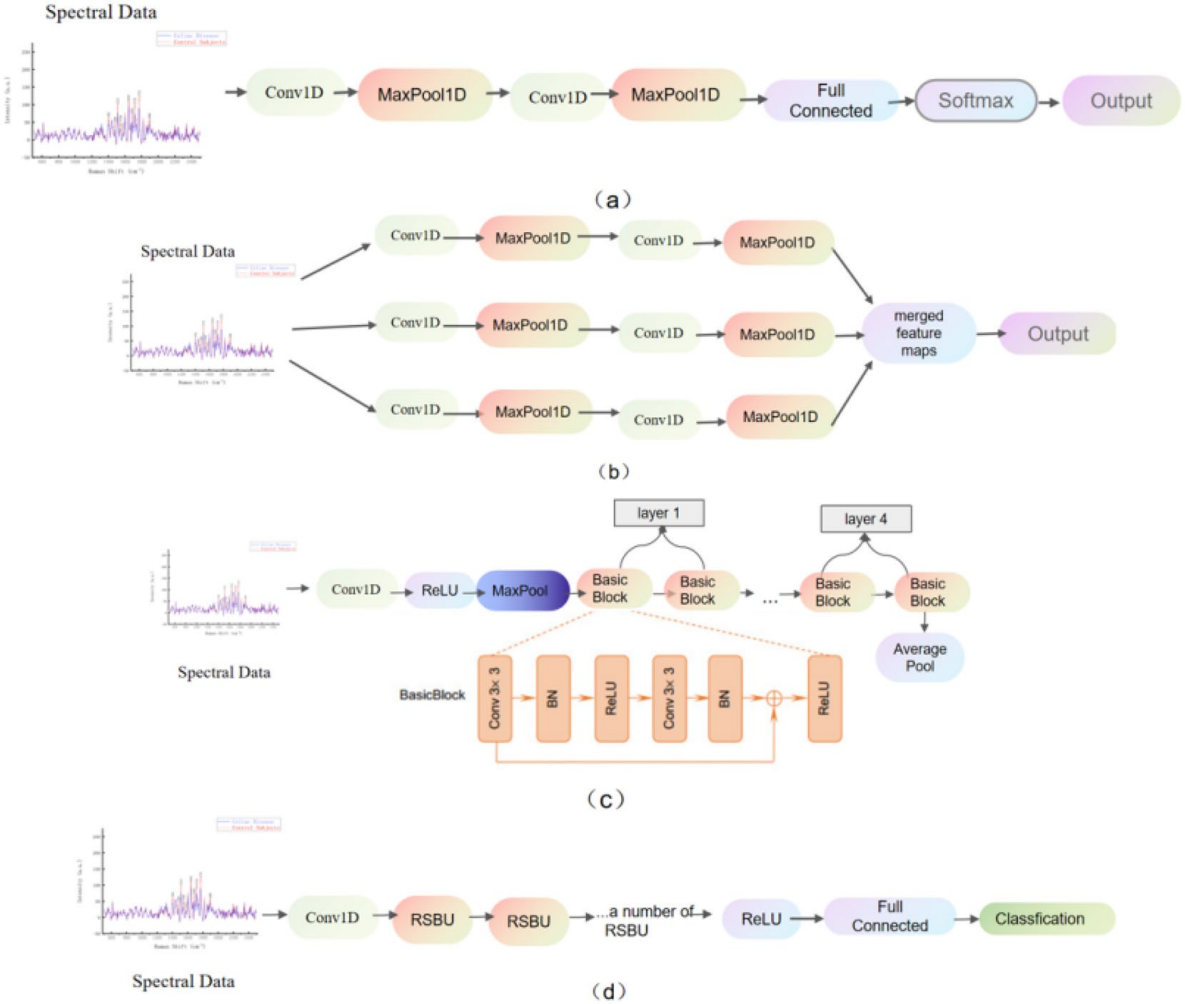
Structure of (**a**) CNN; (**b**) MCNN; (**c**) ResNet; (**d**) DRSN.

Convolutional Neural Network (CNN) is a deep feedforward neural network with features such as local connections and weight sharing. As one of the representative algorithms of deep learning, CNN has significant advantages in complex machine learning problems such as image classification, computer vision, natural language processing^24–27^, making it one of the most widely used models. The components of CNN include basic input and output layers, as well as convolutional layers, pooling layers, and fully connected layers^28^. The convolutional layer is used to extract different features of the input data, which may only be able to extract some low-level features. Most convolution operations can iteratively extract more complex features from low-level features. Then, the pooling layer is used to reduce the dimensionality of the features, achieving feature invariance. As is shown in Fig. 8a, after multiple convolution and pooling operations, all local features are combined into global features in the fully connected layer. In this experiment, the CNN model mainly includes four Conv1D layers with 32, 64, 64, and 32 filters, as well as 2 neurons. A Dropout layer is added after each Dense layer to prevent the problem of model overfitting.

**Figure 9:**
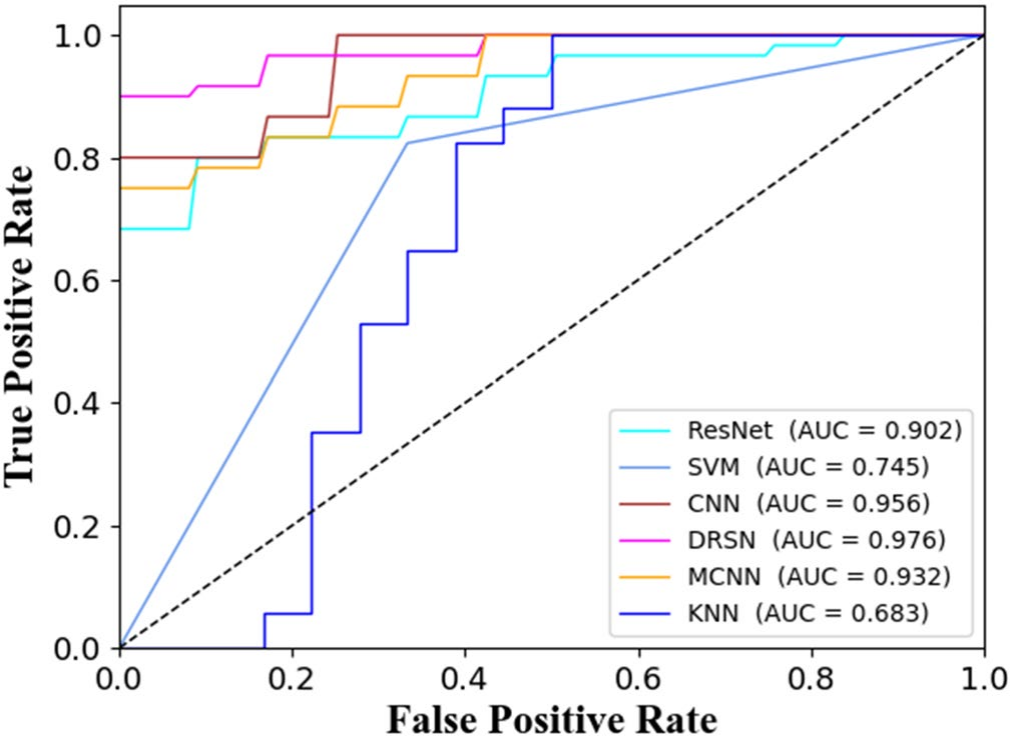
ROC curve of CNN, MCNN, ResNet, DRSN, SVM, KNN.

The ROC curve of the CNN is shown in Fig. 9. Compared to machine learning models, CNN shows improvement in classification accuracy, specificity, and sensitivity. However, CNN still makes errors in recognizing a considerable number of samples.

#### Multi-scale convolutional neural network (MCNN) evaluation

MCNN is a simple yet effective multi-scale convolutional neural network that can map the input to its corresponding density map^29^. MCNN has stronger universality for input information. By using filters of different sizes with different receptive fields, the features learned by convolutional neural networks at different scales have stronger adaptability due to the perspective effect. The MCNN used in this experiment consists of Conv1d layers, LeakyReLu layers, pooling layers, and Conv1d layers. As is shown in Fig. 8b, three convolutional layers are used, with 16, 32, and 64 filters, and kernel sizes of 4, 8, and 16, respectively. The stride is 1, and “same” padding is used. MCNN outperforms the CNN model in accuracy, and the model's runtime is similar to CNN. The ROC curve of MCNN is shown in Fig. 9. From the confusion matrix, it can be seen that MCNN is more powerful in classifying positive samples, which is crucial for the diagnosis of celiac disease.

#### Evaluation of deep residual network (ResNet)

ResNet, as a powerful deep neural network structure, has been widely applied to disease assessment tasks^30^. Its design of residual learning makes the network easier to train and enables deeper feature exploration in images. In disease assessment, ResNet can learn complex features and patterns in medical images, thereby improving the accuracy and robustness in disease diagnosis. The ResNet used in this experiment consists of multiple convolutional blocks, each including a convolutional layer and a batch normalization layer. It also incorporates multiple residual connection blocks, each containing two convolutional blocks and possible convolutional layers for shortcut connections. Dropout layers are added after each residual connection block to prevent overfitting (Fig. 8c). The ResNet model can capture deep features in images and identify potential pathological information. Its structure of direct connections between layers enables better information transmission, alleviating the vanishing gradient problem, and reducing the risk of overfitting. However, its performance on celiac disease spectral data is not superior to that of convolutional neural networks. The ROC curve of ResNet is shown in Fig. 9.

#### Evaluation of deep residual shrinkage network (DRSN)

The Deep Residual Shrinkage Network (DRSN), as a deep learning model, is particularly suitable for features related to noise. It effectively addresses noise and redundant information in spectra, enhancing its learning and feature extraction capabilities for disease features^31^. Built upon ResNet, DRSN introduces improvements by setting a threshold for each channel and incorporating two fully connected layers. As is shown in Fig. 8d, the second fully connected layer outputs neurons equal to the number of input feature map channels, and each neuron undergoes sigmoid activation. DRSN demonstrates significant advantages in handling spectral data^32^, as its residual block structure facilitates deeper exploration of disease features in plasma spectra. Additionally, the introduced shrinkage mechanism effectively suppresses noise in spectral data, enhancing the model's robustness.

By training on celiac disease and healthy control plasma samples, DRSN can learn spectral features related to the disease, achieving precise extraction of potential biomarkers. The design of its network structure allows information to flow between different levels, enabling the model to better capture complex relationships in plasma spectra. Moreover, DRSN's shrinkage mechanism helps reduce redundant information, improving the signal-to-noise ratio of spectral signals. The ROC curve of DRSN is shown in Fig. 9.

### Classification results

Validation results for the CNN, MCNN, ResNet, and DRSN models show that the CNN and MCNN models perform well on the training and validation sets, with accuracies reaching 92.31% and 90.76%, respectively. However, the CNN's specificity is suboptimal at only 85.71%. ResNet exhibits the poorest performance across all metrics, with an accuracy of only 80.23% and specificity of only 68.57%, all have associated 95% confidence interval variance bands. In contrast, the DRSN model outperforms CNN, MCNN, and ResNet in accuracy, specificity, sensitivity, and precision. A crucial factor is the enhanced generalization capability of DRSN in combating noise. And we also compared these models with SVM and KNN. To increase the credibility of the experimental results, this study calculated five evaluation metrics, namely the Area Under the Curve (AUC) of the Receiver Operating Characteristic (ROC) curve, accuracy, sensitivity, specificity, and precision. Table 3 presents the evaluation metrics for the test sets of the four models after five-fold cross-validation.

**Table 3:** Raman spectral model classification results.

Model	AUC %	Accuracy%	Sensitivity%	Specificity%	Precision%
SVM	74.50	74.28	82.35	66.67	80.28
KNN	68.30	80.23	55.29	80.00	72.55
CNN	95.60	92.31	100	73.33	84.28
ResNet	90.20	80.23	100	73.33	84.01
DRSN	97.60	95.00	100	99.13	92.14
MCNN	96.97	90.76	100	80.00	86.67

## Discussion

This study utilized Raman spectroscopy to acquire plasma spectra from patients with celiac disease, revealing differences in the expression of proteins, lipids, amides, and amino acids compared to normal plasma. These differences arise from substantial variances in cellular function and plasma structure between celiac disease and normal plasma cells^33,34^. Leveraging these expression disparities is advantageous for establishing models used to evaluate the extent of differences between celiac disease plasma and true control plasma for classification purposes.

The Raman spectra data used in this study consisted of normal plasma data and celiac disease data, showcasing significant differences in the characteristic peaks of celiac disease plasma compared to normal plasma. Therefore, valuable information can be extracted from plasma Raman spectra data. Overall, among the deep learning models, the Deep Residual Shrinkage Network (DRSN), adept at handling noise to enhance signal-to-noise ratio, demonstrated higher accuracy. The proposed deep learning models, in conjunction with celiac disease Raman spectra data, advance technology in the Raman spectroscopy field and enrich diagnostic approaches for celiac disease.

In this study, we found that the Raman spectra of celiac disease and the healthy control group share most common peaks. To reduce noise, we applied Fourier transform for low-frequency component removal. Additionally, to overcome the drawbacks of low signal-to-noise ratio in Raman spectra, which can lead to low diagnostic performance, we established a Raman spectroscopy diagnostic model based on the Deep Residual Shrinkage Network (DRSN). CNN, ResNet, MCNN, and DRSN achieved high accuracy in disease diagnosis by extracting multiscale features from spectral data. Through a comparative analysis of the four deep learning models, this study observed a gradual improvement in the classification efficiency of celiac disease and healthy control group data, from simple two-layer convolution to complex multilayer convolution, and then to parallel multiscale convolution. The DRSN model effectively alleviated spectral noise issues and exhibited efficient classification performance. It successfully classified celiac disease with an accuracy of 92.3%, sensitivity and specificity both reaching 99.1%. From the analysis results, there were no significant differences in the content of proteins, fatty acids, and phospholipids in the plasma of celiac disease patients and the healthy control group. This may be related to the biological behavior of the disease. Deep learning models, by extracting these differing features, provide a theoretical basis for effective diagnostic classification.

The DRSN model successfully suppressed noise in Raman spectra through the introduction of deep residual shrinkage blocks, enhancing the model's generalization performance. Subsequently, we further explored the impact of scaling coefficients and soft thresholding operations in DRSN on model performance, demonstrating the crucial role of these mechanisms in enhancing the model's robustness and noise resistance. Moreover, there is a need for analysis on the interpretability of the model by visualizing the activation values and feature maps of deep learning models. This would aid in understanding the model's focus on different Raman peaks in the process of discriminating celiac disease, providing insights for further research.

In clinical research, it is crucial to include as much information as possible about patient participants. Raman spectroscopy is holistic and not targeted towards individual analytes, thus the entire biological fluid influences the detected signal. Therefore, we supplemented patient information such as age, gender, geographic location, etc. allowing for further investigation and comparison with future studies. This information can be included in the Table 4.

**Table 4:** The relevant patient information.

Order	Group	Gender	Age	Ethnicity	Course of illness	BMI
1	CeD	Male	20	Uyghur	3 years	27.16
2	CeD	Female	24	Uyghur	0.17 years	23.24
3	CeD	Female	26	Han	3 days	32.27
4	CeD	Female	32	Kazakh	0.5 years	19.84
5	CeD	Female	33	Kazakh	1 year	20.07
6	CeD	Female	39	Uyghur	4 years	17.19
7	CeD	Female	40	Kazakh	10 years	20.61
8	CeD	Female	40	Kazakh	15 years	25.78
9	CeD	Female	40	Kazakh	0.5 years	20.59
10	CeD	Female	41	Uyghur	1 month	20
11	CeD	Female	42	Uyghur	2 years	14.5
12	CeD	Female	42	Uyghur	7 years	18.3
13	CeD	Female	43	Uyghur	3.5 years	24.03
14	CeD	Female	44	Uyghur	5 years	22.86
15	CeD	Male	44	Kazakh	0.17 years	19.62
16	CeD	Female	45	Kazakh	0.5 years	19.49
17	CeD	Female	45	Kazakh	0.5 years	19.49
18	CeD	Male	49	Kazakh	0.5 years	23.88
19	CeD	Female	50	Uyghur	3 years	24.22
20	CeD	Female	50	Kazakh	1 years	17.20
21	CeD	Female	51	Han	2 years	21.88
22	CeD	Male	51	Kazakh	3 months	22.20
23	CeD	Male	55	Han	1 years	27.16
24	CeD	Male	56	Uyghur	1 week	17.99
25	CeD	Female	56	Kazakh	15 years	25.78
26	CeD	Female	58	Uyghur	0.17 years	22.27
27	CeD	Female	59	Kazakh	10 years	19.53
28	CeD	Male	60	Uyghur	3 years	24.45
29	CeD	Male	46	Uyghur	1 years	18.93
30	Control	Male	22	Uyghur		19.57
31	Control	Female	26	Uyghur		19.57
32	Control	Female	28	Han		17.30
33	Control	Female	33	Kazakh		19.82
34	Control	Female	35	Kazakh		26.60
35	Control	Female	38	Kazakh		21.01
36	Control	Female	40	Uyghur		25.08
37	Control	Female	40	Kazakh		21.95
38	Control	Female	41	Kazakh		28.34
39	Control	Female	42	Uyghur		24.37
40	Control	Female	42	Uyghur		16.96
41	Control	Female	42	Uyghur		20.55
42	Control	Female	42	Uyghur		16.77
43	Control	Female	42	Uyghur		24.03
44	Control	Female	44	Kazakh		23.03
45	Control	Female	44	Kazakh		24.09
46	Control	Male	45	Kazakh		23.14
47	Control	Female	48	Uyghur		23.23
48	Control	Female	48	Kazakh		25.61
49	Control	Male	50	Kazakh		21.64
50	Control	Female	52	Han		32.20
51	Control	Male	52	Kazakh		22.60
52	Control	Female	55	Kazakh		29.67
53	Control	Male	56	Han		24.84
54	Control	Female	57	Uyghur		23.41
55	Control	Male	58	Uyghur		20.43
56	Control	Male	58	Uyghur		24.61
57	Control	Male	52	Uyghur		23.64
58	Control	Male	26	Han		22.55
59	Control	Female	44	Uyghur		20.55

In conclusion, this study provides strong empirica support for combining Raman spectroscopy and deep learning models for celiac disease diagnosis. Future work could expand the sample size, consider multicenter data to verify the model's robustness, and delve deeper into exploring the interpretation and discovery of potential biomarkers by deep learning models for celiac disease. This is crucial for advancing the translational application of spectroscopic diagnostic technology in clinical settings.

## Conclusion

In the face of the limited information extracted from conventional plasma assays, the challenges of effectively distinguishing the highly similar spectra exhibited by celiac disease, which are difficult for the human eye to discern, and the presence of noise interference in spectral data, this study further confirmed the high efficiency of deep learning networks for extracting multi-scale features. Specifically, comparing the classification performance of four deep learning models on plasma Raman spectra of celiac disease patients and healthy control groups, this study demonstrates the effectiveness of deep learning networks for feature extraction.

For the DRSN model, its end-to-end learning approach allows direct learning of feature representations from raw spectral data without the need for manually designing feature extractors. This reduces the need for feature engineering and enhances the model's automation. The network structure of DRSN allows information to flow between different levels, enabling the model to learn multi-scale features. This is crucial for capturing spectral information at different levels and aids in comprehensive understanding and extraction of disease-related biomarkers. Through extracting multi-scale and multi-level features from spectral data, DRSN achieves non-invasive, rapid, and low-cost identification of celiac disease patients and healthy control group data.

Ultimately, our research results indicate that DRSN has achieved significant success in the classification and diagnosis of celiac disease and healthy controls. By comparing the performance of different models, our conclusion is that adopting DRSN can effectively improve the accuracy and robustness of disease diagnosis. Furthermore, due to its efficiency in handling spectral noise, DRSN excels in spectral data processing and disease monitoring tasks. It serves as a powerful tool for accurately and efficiently extracting disease features and conducting spectral data analysis. This valuable experience and guidance contribute to future research in celiac disease classification within the spectroscopy measurement field.

## Data Availability

The datasets generated and analyzed during the current study are not publicly available due to data privacy laws, but are available from the corresponding author on reasonable request.
